# Improved prognostic diagnosis of systemic lupus erythematosus in an early stage of disease by a combination of different predictive biomarkers identified by proteome analysis

**DOI:** 10.1186/1878-5085-5-5

**Published:** 2014-03-20

**Authors:** Maximilian Boenisch, Rebecca Hurst, Susanna Huber, Jadranka Koehn, Kurt Krapfenbauer

**Affiliations:** 1 University of Vienna, Universitätszentrum II, Althanstrasse 14, Vienna 1090, Austria; 2 University of Manchester, Manchester M13 9PL, UK; 3 Medical University of Vienna, Waehringer Guertel 18-20, Vienna 1090, Austria

**Keywords:** Lupus nephritis, Panel of predictive biomarker, Systemic lupus erythematosus, MRL-lpr/lpr mouse model

## Abstract

**Background:**

Since the original characterizations of the pathological features defining glomerulonephritis in systemic lupus erythematosus (SLE) were reported, numerous studies have linked the development of pathology to the abnormal expression of protein in urine. The determination of proteinuria is important and necessary; however, this alone is not predictive enough to confirm a suspected diagnosis, especially in an early state of disease when symptoms are not yet observed. Furthermore, several studies have already highlighted the pitfalls of proteinuria both as a clinical prognostic marker and as a factor predicting the progressive loss of renal function. Therefore, the identification of more accurate and predictive biomarkers is urgently needed. To address this, comparative urinary and kidney profiling was performed in the MRL-lpr/lpr mouse as a model of lupus tubulointerstitial nephritis and lupus glomerulonephritis corresponding to SLE in humans.

**Results:**

Tamm-Horsfall glycoprotein (THG; uromodulin) and beta2-microglubulin (β2M) were identified as immune process-related molecules in the urine and kidney of the MRL-lpr/lpr mouse model. Furthermore, we show that the combinatory expression profile of THG and β2M as biomarkers, normalized by the proteinuria level, is more predictive than proteinuria determination alone. Data were confirmed by comparative urinary profiling of SLE in mice by Western blot and quantitative polymerase chain reaction (qPCR) analysis.

**Conclusion:**

Based on our results, we are able to diagnose SLE in the MRL-lpr/lpr mouse in a very early state of disease, when the proteinuria level alone is not able to confirm a suspected diagnosis. The pre-validation of our urinary biomarkers is associated with clinical outcomes of glomerulonephritis in humans and merits additional investigation. Further conformations of our predictive biomarkers in the urine of SLE patients in the course of a clinical study are still ongoing.

## Overview

Systemic lupus erythematosus (SLE) is a chronic, inflammatory, systemic autoimmune disease in humans of unknown origin. It is a multisystem disease characterized by a strong female predilection, a diversity of autoantibodies of which those against nuclear antigens typically predominate, immune complex formation and deposition, endothelial cell and complement activation and leucocyte emigration and activation [1]. It has a prevalence of between 2 and 7.6 cases per 100,000 individuals [2], depending on ethnicity, and causes significant morbidity and increased mortality, particularly for patients with renal or central nervous system involvement [3]. The immunopathology of lupus is considered to result from loss of peripheral tolerance, production of autoantibodies and subsequent autoantibody-mediated tissue destruction. Lupus nephritis (LN), which occurs in up to two thirds of patients with SLE at some stage of their illness [4], is believed to result mostly from renal deposition of autoantibodies and immune complexes [5], which in turn triggers an acute inflammatory response characterized by activation of infiltrating leucocytes and renal parenchymal cells. This activation is accompanied by the production of cytokines and growth factors. End-stage renal disease (ESRD) is preceded by a chronic phase with excessive deposition of collagen and other extracellular matrix macromolecules [6]. The etiology of this disease is still unknown, but evidence supports a strong genetic predisposition as well as a role for stochastic and environmental factors.

The diagnosis of SLE is made based on the presence of 4 of 11 criteria set out by the American College of Rheumatology (ACR). These include kidney biopsy and function tests, e.g. proteinuria. Kidney biopsy is essential for LN diagnosis, but this is clearly not a simple procedure and is associated with an inherent risk. Some of the routine laboratory parameters such as urinary sediments, serum level of DNA-anti-DNA immune complexes, and complements are also helpful in determining the activity of LN [7-9]. However, sampling is not simple due to invasive procedures and the frequent need for repeat sampling to confirm initial values. Besides, fluctuations of these parameters frequently occur during the development of glomerular as well as tubulointerstitial pathology. The diagnosis of LN and other kidney-specific diseases is therefore often made at a late state of disease, when first symptoms are observed and medication is already required. Furthermore, there is an urgent need to identify better predictive markers for kidney function that are more sensitive or more reliable than the markers currently used. A robust and alternative determination of the amount of immune process-related biomarkers in urine would thus be an effective alternative way to evaluate the severity of renal inflammation in LN. Following the recommendations of the *EPMA* white paper [10,11] on developing integrative medical approaches, our predictive panels of biomarkers in the urine of SLE patients in the course of a clinical study are still ongoing and data will be published in an upcoming *EPMA* Journal.

## Methods

### Scientific rational for using MRL-lpr/lpr mice as a model of human SLE

Several animal models, manifesting phenotypes observed in SLE, have been identified in nature or generated in the laboratory. These models generally present physiological alterations observed in human patients and can be used as important tools for genetic, clinical and histopathological studies for lupus tubulointerstitial nephritis and lupus glomerulonephritis. The MRL-lpr/lpr mouse model is the most widely used animal model for tubulointerstitial nephritis and lupus glomerulonephritis corresponding to SLE in humans. It is a good genetic and biochemical model characterized by glomerular immune complex deposits and proliferative glomerulonephritis, progressing to glomerulosclerosis and kidney failure, and is therefore a well-accepted mouse model for clinical trials of LN.

The animals were maintained conventionally under standard conditions (22°C ± 1°C, 55% ± 5% relative humidity, 10 changes of fresh air, 12:12-h day/night cycle) in groups in type III Makrolon® cages with sawdust bedding (Bayer MaterialScience LLC, Sheffield, MA, USA). Standard laboratory chow (SNIFF® R/M-H diet for mice and rats, Research Diets, Uden, The Netherlands) and drinking water from the municipal supply were given *ad libitum*. Mice were housed in groups during the treatment period.

### Statement on animal welfare

Studies described in this report were performed according to a notification (§9, Österreichisches Tierversuchsgesetz) approved by the governmental authority (Landesregierung Wien, Magistratsabteilung 58).

### Animal experimental conditions

Mice were divided into three groups of mice weighing between 25 and 35 g. The treatment started at 10 weeks of age and continued daily for 9 weeks. Groups received vehicle control or cyclophosphamide (Sigma C-0768, St. Louis, MO, USA). The treatment schedule is described in Table 1. The CpG oligo-ODN 1668 5′ TCG ATG ACG TTC CTG ATG CT 3′ was used as a disease accelerator.

**Table 1:** THG oligonucleotide primers and probes

**Group**	**Strain**	**Treatment**	**Dose**	**Route**	**Concentration at delivery (mg/ml)**	**Delivery volume**
1	MRL-lpr/lpr	Vehicle	bid	p.o.	-	10 μl/g
2	MRL-lpr/lpr	Cyclophosphamide	35 (mg/kg/week)	i.p.	5	7 μl/g
3	MRL-lpr/lpr	CpG-ODN	40 μg on alternate days	i.p.	-	100 μl

According to [12].

#### Disease monitoring

MRL-lpr/lpr mice were monitored weekly throughout their disease course for body weight and proteinuria. If they became moribund, mice were sacrificed by cervical dislocation. Moribund animals usually demonstrated signs of severe nephritis, such as persistent proteinuria and body weight loss. At sacrifice, the kidney was excised and fixed. Proteinuria and kidney histopathology severity scores were determined using the protocols described below.

### Evaluation of kidney disease

#### Proteinuria

Twenty-four-hour urine samples were collected from each animal at regular intervals from 10 weeks of age until the end of the experiment. Urine protein levels were scored. For proteinuria determination, two different assay methods were selected and performed according to the manufacturer's protocol: the BCA protein assay (Pierce, Cat. No. 23227, Thermo Fisher Scientific, Waltham, MA, USA) and the Bradford assay (Bio-Rad, Cat. No. 500-0006, Bio-Rad Laboratories, Hercules, CA, USA) [13]. Based on the higher stability and enhanced reproduction of the results, the Bradford assay was commonly used.

#### Kidney histopathology

Kidney sections from Bouin's solution-fixed (HT10-1-32, Sigma, Vienna, Austria) paraffin-embedded material were stained in periodic acid-Schiff (PAS) reagent (Cat # 21-1308021C, Bio-Optica, Milan, Italy). Histologic examination of sections was carried out in a blind manner, and the severity of kidney disease was defined using the activity/chronicity index according to [14]. Table 2 outlines the specific histologic features of this renal pathology semi-quantitative scoring system. Composite scores derived from the sum of scores for individual active lesions (activity index) and chronic, irreversible lesions (chronicity index) were calculated. Two active lesions, fibrinoid necrosis and cellular crescents, were each arbitrarily weighted by a factor of 2 on the assumption that these features are disproportionately more ominous than the other active lesions.

**Table 2:** A/C scoring criteria

	**Activity index**	**Chronicity index**
Glomerular abnormalities	Cellular proliferation	Glomerular sclerosis
	Fibrinoid necrosis, karyorrhexis^a^	Fibrous crescent
	Cellular crescents^a^	
	Hyaline thrombi, wire loops	
	Leucocyte infiltration	
Tubulointerstitial abnormalities	Mononuclear cell infiltration	Interstitial fibrosis
		Tubular atrophy

Scoring from 0 to 3, where 0, absent; 1, mild; 2, moderate; 3, severe. ^a^Weighted by a factor of 2. The maximum score of the activity index is 24, and that of the chronicity index is 12.

### Proteomics procedure

#### Urine collection

In proteomics, urine collection tubes should always include an appropriate amount and composition of protease inhibitors to avoid protein degradation. After urine collection, delays in analyzing the samples can result in artefacts, and thus, it is important to handle the samples quickly. Note that if a sample cannot be analyzed immediately, it should be frozen (-80°C). The time interval between collection and analysis should be as short as possible. A delay in this handling step could have a high impact on the urine status and protein pattern. Changes that can occur with time after collection include protein degradation caused by proteases in urine, decreased clarity due to crystallization of solutes, rising pH, loss of ketone bodies, loss of bilirubin, cell lysis leading to additional proteins in samples, and overgrowth of contaminating microorganisms. For this reason, the main challenges in working with urine for biomarker discovery are in the standardization of urine sample collection, storage, shipment (if necessary), enrichment of potential markers with low abundances and quantification of the excretion rate from a single marker. In the course of our project, mouse urine samples from the whole day were collected in 0.5-ml tubes including a protease inhibitor cocktail (cOmplete, Roche Diagnostic, 11 697 498 001, Basel, Switzerland), immediately frozen at -80°C and kept frozen until use. Samples were handled and stored as described [15].

#### Kidney sample homogenization

Whole kidney tissue samples were suspended in sucrose buffer consisting 20 mM HEPES (pH 7.5), 320 mM sucrose, 1 mM ethylenediaminetetraacetic acid (EDTA), 5 mM DTE, 1 protease inhibitor cocktail tablet (Roche Diagnostic, Art. Nr. 11 697 498 001)/50 ml homogenization buffer, 1 mM phenylmethanesulfonyl fluoride (PMSF), 0.2 mM Na_3_VO_3_ and 1 mM NaF using a glass-Teflon potter as a homogenizer. After 10 strokes at 4°C, the suspension was centrifuged at 800 × *g* for 10 min at 4°C to sediment the non-suspended material. To receive the fractions enriched with microsomal, mitochondrial and cytosolic proteins, the supernatant was further centrifuged at 10,000 × *g* for 15 min at 4°C and then at 100,000 × *g* for 1 h at 4°C. The cell debris of the mitochondrial and microsomal fractions was suspended in 0.5 ml of a sample buffer consisting of 40 mM Tris, 7 M urea, 2 M thiourea, 4% CHAPS, 10 mM 1,4-dithioerythrol, 1 mM EDTA and a mixture of protease inhibitor, 1 mM PMSF and 1 protease inhibitor cocktail tablet (Roche Diagnostic, Art. Nr. 11 697 498 001)/50 ml homogenization buffer. The supernatant was further centrifuged at 100,000 × *g* for 1 h to sediment the undissolved material. The protein content in the supernatant was determined by a Bradford reaction performed as per the manufacturer's protocol. The protein concentration is normally approximately 8–12 mg/ml for the main fraction.

#### Electrophoresis

Samples of the mitochondrial, microsomal and cytosolic fractions were desalted by using membrane filter tubes (Millipore, Art. No. UFV4BGC25, Billerica, MA, USA), and 2.0 mg was applied on immobilized pH 3–10 non-linear gradient strips (Bio-Rad, Hercules, CA, USA) at both the basic and acidic ends of the strips. The proteins were focused at 200 V, after which the voltage was gradually increased to 3,000 V with 3 V/min. Focusing was continued at 3,000 V for additional 12 h. The second-dimension separation was performed on 12% non-linear gradient, polyacrylamide gels (Serva, Heidelberg, Germany, and other reagents for the polyacrylamide gel preparation from Bio-Rad, Hercules, CA, USA).

#### Staining and destaining

After protein fixing with 50% (*v*/*v*) methanol containing 5% (*v*/*v*) phosphoric acid for 12 h, the gels were stained with colloidal Coomassie blue (Novex, San Diego, CA, USA) for further 24 h. The gels were destained with water and scanned in a Molecular Dynamics Personal densitometer (Amersham Pharmacia Biotechnology, Amersham, UK). The images were processed using Photoshop (Adobe) and PowerPoint (Microsoft) software. Protein spots were quantified using the ImageMaster 2D Elite software (Amersham Pharmacia Biotechnology).

#### Identification of the proteins by PepMap ESI-MS

Electrospray ionization mass spectrometry (ESI-MS) analysis was performed as described [16] with minor modifications. Briefly, spots were excised, destained with 30% (*v*/*v*) acetonitrile in 0.1 M ammonium bicarbonate and dried in a SpeedVac evaporator (Thermo Savant, Inc., Holbrook, NY, USA). The dried gel pieces were reswollen with 5 μl of 3 mM Tris-HCl (pH 8.8) containing 50 ng trypsin (Promega, Madison, WI, USA), centrifuged for 1 min and left at room temperature for about 12 h. After digestion, 5 μl of water was added, followed 10 min later by 4 μl 50% acetonitrile, containing 0.3% trifluoroacetic acid, centrifuged for 1 min, and the content was vortexed for 20 min. Ten microlitres from the separated liquid was used to perform the ESI-MS. Calibration was internal to the samples. The peptide masses were matched with the theoretical peptide masses of all proteins from all species of the SWISS-Prot database. For protein search, monoisotopic masses were used and a mass tolerance of ≈ 0.0075% was allowed. The protein search was performed with software which is similar to the Peptident software on the ExPASy server (http://expasy.hcuge.ch/sprot/peptident.html).

#### Validation by Western blot procedure

The protein content of each sample was determined by the method of Bradford [13]. For Western blot analysis, equal amounts of protein (15 μg) were separated by electrophoresis on a 10% TB gel (NuPAGE, Invitrogen, Life Technologies, Carlsbad, CA, USA) using XCell II and Western transfer using the XCell II blot module, transferred to a nitrocellulose membrane (Bio-Rad) following the manufacturer's guidelines and description. An affinity-purified polyclonal rabbit antibody, raised against a 105-kDa portion of human Tamm-Horsfall glycoprotein (THG) was purchased from Abcam (Cambridge, UK). After transfer, membranes were blocked for 14 h in a phosphate-buffered saline-Tween 20 (PBS-T) blocking/wash buffer (PBS adjusted to pH = 7.5, 5% non-fat dried milk powder and 0.05% Tween 20). After blocking, the membranes were immunoblotted with polyclonal rabbit anti-THG (1:5,000 (*v*/*v*) PBS blocking buffer) for 1 h at 25°C. After 3 × 15 min of washing with PBS, the bound antibodies were visualized by HRP-conjugated goat anti-rabbit IgG (Pierce) secondary antibody (1:10,000 (*v*/*v*) PBS blocking buffer), and after further 3 × 15 min of washing, by a chemiluminescence detection method (Pierce). Exposed and developed films (Pierce) were scanned, and internal densities of THG and THG-immune reactive bands were calculated by the ImageMasterII software program (GE Healthcare, Little Chalfont, UK).

### Genomics procedure

#### RNA preparation

Whole kidney samples were homogenized by using a bead-based homogenizer (Precellys 24, Bertin Technologies, Montigny-le-Bretonneux, France), which allows the extraction of several samples simultaneously. Each tissue occupied a separate sterile plastic tube to avoid sample carry-over and cross-contamination. Homogenization was completed within 30 s for 5,600 rpm in pre-cooled tubes. Total RNA was extracted by the TRIzol method (Invitrogen) with some modification as described in the classic TRIzol/1-bromo-3-chloropropane/water extraction protocol [17]. RNA concentration and purity were determined with a spectrophotometer (BioSpectrometer, Eppendorf, Hamburg, Germany) by calculating the ratio of optical density at 260 and 280 nm.

#### Validation of THG by RT-qPCR

Primer and probe sequences for THG (forward primer: CCCTGAGTGCAATCTGGCTTA, reverse primer: GCAATCTTCATCTACCCTGCATT, probe: TGCACCGATCCTAGTTCCGTGGAGG) were selected using the Primer Express™ software (Applied Biosystems, Foster City, CA, USA). To avoid genomic DNA, amplification primers were located in different exons. Primer and probes for the housekeeping gene glyceraldehyde-3-phosphate dehydrogenase (GAPDH) were obtained from Applied Biosystems as a ready mix (TaqMan Gene Expression Assay, Order Nr. 4400239).

cDNA synthesis was carried out using the iScript™ cDNA Synthesis Kit (Bio-Rad) according to the manufacturer's instructions to transcribe 300 ng RNA in a total volume of 30 μl.

Using 1 μl cDNA as a template, real-time quantitative polymerase chain reaction (RT-qPCR) was performed in a total volume of 20 μl with a final concentration of 400 nM forward/reverse primers and 200 nM TaqMan probe for THG or 1:20 dilution of GAPDH TaqMan Gene Expression Assay using the Eppendorf Real Master Kit, according to the manufacturer's protocol in an Eppendorf Mastercycler ep realplex gradient S. An initial denaturation for 2 min at 95°C was followed by 40 cycles with 10 s at 95°C and 45 s at 60°C. PCR was performed in a 96-well format (PCR plates, twin.tec, skirted, Eppendorf) in technical duplicate. Medium *C*_T_ values were used for relative quantification compared to the GAPDH gene according to the formula of the ΔΔ*C*_T_ method [18]. Expression levels of THG were normalized to the average of GAPDH.

### ELISA procedure for measurement of beta2-microglobulin according to the EIA method

#### Preparation of standard rat/mouse beta2-microglobulin solution

For beta2-microglobulin determination, a corresponding protein standard from a kit (Mitsubishi Chemical Safety Institute Ltd., Cat. No. PRH111, Minato-ku, Tokyo, Japan) was used. For accurate preparation, 2.0 ml of distilled or deionized water was added into the vial containing the standard rat beta2-microglubulin (β2M; Catalogue Nr. PRH111, Mitsubishi Chemical Safety Institute Ltd.), providing a concentration of 20 ng/ml. Serial dilutions were prepared to produce standard concentrations of 10, 5, 2.5, 1.25, 0.63 and 0.31 ng/ml. For 0 ng/ml, the sample diluent buffer was used.

#### Preparation of test samples

Samples from mouse urine were collected in the presence of a protease inhibitor (cOmplete, Roche Diagnostic). The pH values of the test samples were adjusted to 6.5–8.0, since β2M is completely denatured in 2 h when the pH is 4.0 and the temperature is 37°C. When kept stored at 4°C, for example, when β2M is measured in serum [15], the urine sample can remain stable for 1 week. If stored below -20°C, it will remain stable for 12 months. To avoid high background and interference from the urine, the samples were diluted into more than 150-fold volume. As the content of β2M in urine is expected to be in the order of micrograms in 1 ml of urine, the test samples were diluted whenever deemed necessary, e.g. 2 ml of the sample diluent buffer was added into 10 μl of urine to dilute it into 201-fold volume.

### Statistics

Results were expressed as means ± standard deviation (SD). Between-group differences were investigated by the non-parametric Mann-Whitney *U* test. Within-group correlations were done with the non-parametric Spearman rank correlation coefficient procedure. The level of significance was set at *P* ≤ 0.05. All analyses were run in duplicate.

## Results

### Detection of THG protein in urine by proteomics

#### Characterization of the MRL-lpr/lpr urine proteome by depletion of impurities by protein precipitation in combination with 2DE

**Figure 1:**
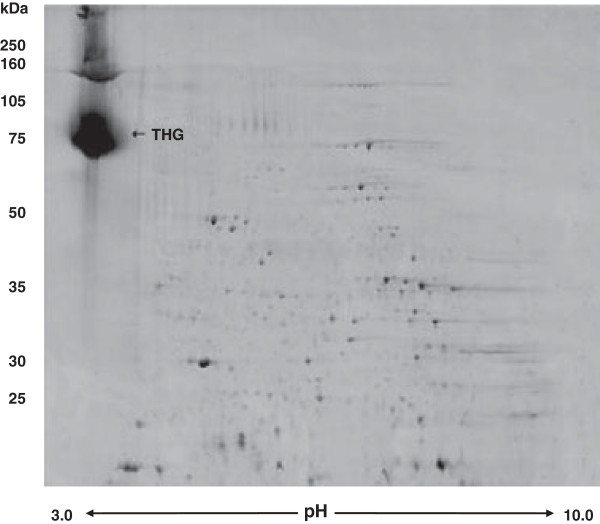
**Proteome identification of Tamm-Horsfall glycoprotein (*THG*) as a biomarker by 2DE-PAGE of mouse urine.**

**Figure 2:**
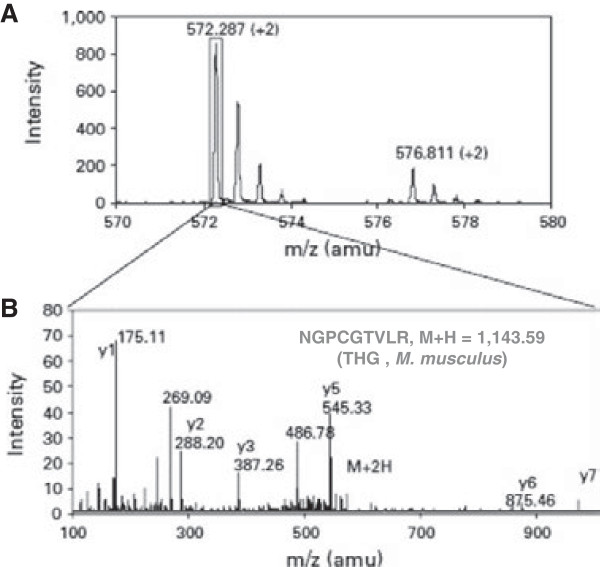
**Identification of THG by peptide fingerprint MS spectra for the THG (uromodulin) peptide NGPCGTVLR.** A single ESI-MS spectrum is shown **(A)**, with the relative quantitation of the protein in the heavy- and light-labelled mixtures obtained by comparing the peak intensities of monoisotopic peaks at *m*/*z* of 576.811 and 572.287, respectively. The peak at *m/z* of 572,287 was select for further MS/MS analysis in the linear modus of the ESI-MS/MS **(B)**. The spectrum obtained was matched to the sequence of NGPCGTVLR-12C9-cICAT from uromodulin (*M. musculus*). Only the y ions are labelled for simplicity.

In urinary proteome analysis, control samples are usually diluted and require additional processes in order to yield enough proteins for further investigation. The application of two-dimensional gel electrophoresis (2DE) to urine requires further steps in sample preparation, including the concentration and isolation of the proteins. Specifically, high concentrations of salts, metabolic wastes, lipids and some small molecules decrease the quality of the 2D gel and must be removed before analysis. Several protocols were developed to overcome these problems and to improve the quality of the urine 2D maps. In our case, we used protein precipitation because it is the most common method to remove contaminants or interfering substances from urine samples. It provides a simple, rapid and highly economic method for concentrating urine of low to intermediate protein content (0.02–0.50 g/l) prior to 2DE analysis. In the first step, proteins were separated by 2DE according to their masses and isoelectric points using pH 3–10 NL IPG strips followed by 12% SDS gels. The gels were stained with colloidal Coomassie blue. The main spot in Figure 1 was excised, after in-gel enzymatic digestion. The tryptic products of the main spot were further analyzed by ESI-MS resulting in the mass fingerprint as shown in Figure 2. The identification was performed by databank search, and the main spot at 95 kDa was mainly identified as THG. Besides THG, the most frequently observed peptides from the main spot were proteolytic products of haemoglobin, including multiple fragments from both the α and β subunits. The existence of these fragments is to be expected as microhematuria, a characteristic finding in lupus nephritis [6] and vasculitis [14].

A single ESI-MS spectrum is shown in Figure 2A. The peak at *m*/*z* of 572.287 was selected for further MS/MS analysis. The spectrum obtained was matched to the sequence from uromodulin (*Mus musculus*). Only the y ions for PMF identification are labelled for simplicity.

### Confirmation of THG expression by Western blot

By comparison of the 2DE-PAGE urinary protein pattern, THG was mainly identified as an upregulated spot in the vehicle- and CpG-treated mice. Images indicate that THG is a useful and good biomarker, which was further confirmed by the Western blot procedure.

**Figure 3:**
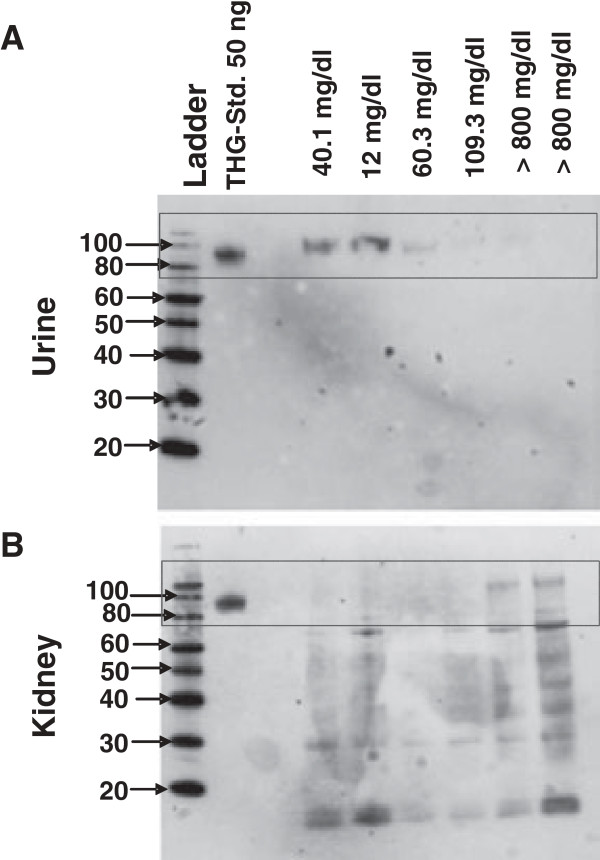
**THG levels in MRL-lpr/lpr mouse urine and kidney samples taken at different time points.** THG levels in **(A)** MRL-lpr/lpr mouse urine and **(B)** kidney samples taken at different time points. THG was detected only in the urine samples of healthy mice and was almost absent in the urine of diseased MRL-lpr/lpr mice. Furthermore, we found a positive correlation of THG levels in the kidneys and a negative correlation of THG levels in the urine with disease level. The reduction of urinary THG is particularly intriguing.

THG was detected only in the urine samples of healthy control mice and was not detectable in any other organs (data are not shown), indicating the organ specificity of anti-THGAb. Compared to the MRL-lpr/lpr mice, a positive correlation of THG level in the kidney with increased severity of the disease was observed as shown in Figure 3.

THG was detected only in the urine samples of healthy mice and was almost absent in the urine of diseased MRL-lpr/lpr mice (Figure 3A) while being detectable in the kidney samples of diseased MRL-lpr/lpr mice (Figure 3B). Furthermore, we found a positive correlation of THG levels in the kidneys and a negative correlation of THG levels in the urine with disease level as determined, e.g. by proteinuria.

**Figure 4:**
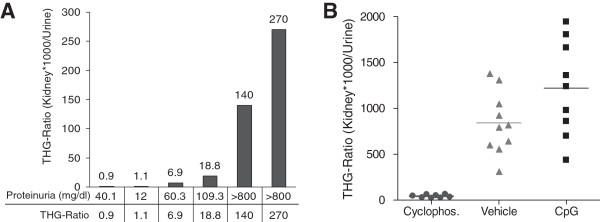
**Ratio of THG levels in the kidney vs. urine of MRL-lpr/lpr mice after treatment.** The ratio of the THG levels in the kidney and urine correlates with disease severity as determined by proteinuria in MRL-lpr/lpr mice **(A)**. The ratio of THG levels in the kidney and urine is significantly reduced in the MRL-lpr/lpr mice protected from SLE by treatment with cyclophosphamide, while the vehicle- or CpG-treated mice show higher THG ratios **(B)**.

The ratio of THG levels in the kidney vs. THG levels in the urine of MRL-lpr/lpr mice treated with cyclophosphamide is significantly lower (*P* < 0.001) than the ratio of the vehicle- or CpG-treated mice (Figure 4). The mean value in each group is shown by a horizontal line.

THG mRNA expression in kidney tissue

**Figure 5:**
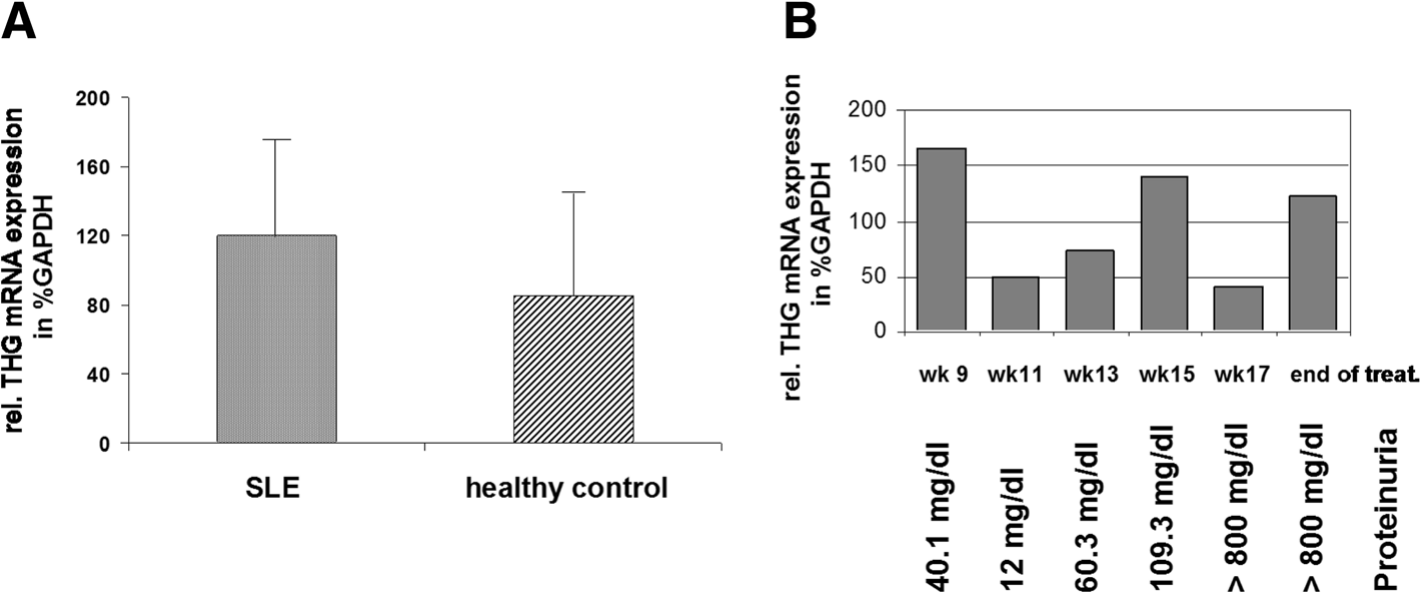
**Relative mRNA expression of THG in MRL-lpr/lpr mice kidneys.** THG and GAPDH mRNA were determined in kidney homogenates from the vehicle-treated mice classified healthy or SLE-diseased based on the proteinuria level. THG mRNA expression was normalized to GAPDH mRNA and calculated in relation to the relative expression of healthy animals **(A)**. Results are representative of two animals in each group with an identical LN score. THG mRNA was detected in kidney samples of the vehicle-treated mice at different time points and correlated to proteinuria **(B)**.

Real-time PCR was performed on cDNA from mRNA isolated from whole kidney homogenates of two healthy control mice with a proteinuria level less than 50 mg/dl and two SLE diseased MRL-lpr/lpr mice with a proteinuria level higher than 100 mg/dl. Changes in mRNA expression of THG from 8- to 19-week-old mice, treated with vehicle, were determined as described in the ‘Genomics procedure’ section. mRNA levels were determined after normalization for GAPDH and are relative to expression in healthy control mice (Figure 5A). Although diseased mice show a slightly higher THG mRNA expression than healthy mice, this difference was not significant. Contrary to the urinary THG protein levels in the vehicle-treated mice (Figure 3), no correlation of THG mRNA levels with age or proteinuria levels can be detected (Figure 5B). These results indicate that mRNA expression does not always correlate directly with changes in protein expression/secretion. The lesser degree of changes in THG mRNA levels than in THG urinary protein may indicate complement-dependent changes in protein expression as a result primarily of inhibition of THG secretion into the urine. Figure 5A is representative of two mice per group obtained with an identical LN score.

Comparison of beta2-microglobulin, THG and proteinuria as disease markers

**Figure 6:**
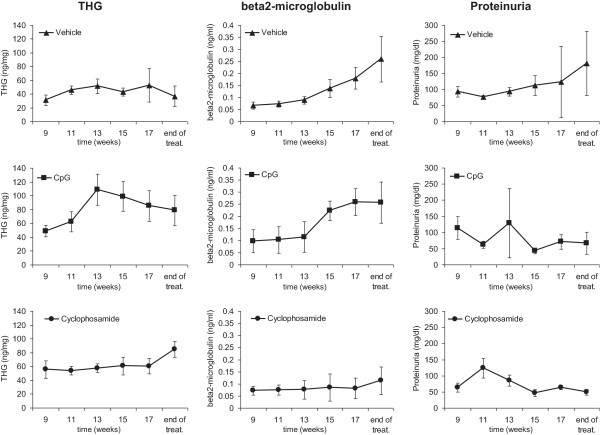
**THG, beta2-microgloblin and proteinuria levels in the urine of MRL-lpr/lpr mice at different time points.** THG, beta2-microglobulin and proteinuria levels were detected in the urine of MRL-lpr/lpr mice treated with cyclophosphamide, CpG or vehicle at different time points. Results show the mean ± SD in each treatment group.

THG, beta2-microglobulin and proteinuria levels were determined at different time points in urine samples of MRL-lpr/lpr mice treated with cyclophosphamide, CpG or vehicle (Figure 6). Results show the mean ± SD in each treatment group. The CpG-treated mice had a significantly higher THG level in urine than the corresponding cyclophosphamide- or vehicle-treated mice towards the end of the treatment. The level of beta2-microglobulin increased significantly with time in mice treated with CpG or vehicle, but not in mice treated with cyclophosphamide. For over 20 years, cyclophosphamide, as an immunosuppressive agent, has been the ‘gold standard’ for the treatment of lupus nephritis. The side effects of this agent are infertility, malignancy, hemorrhagic cystitis and infection, and therefore, other immunosuppressive agents, such as CpG, are preferred for maintaining remission, because of their greater safety.

Predictive potential of beta2-microglobulin

**Figure 7:**
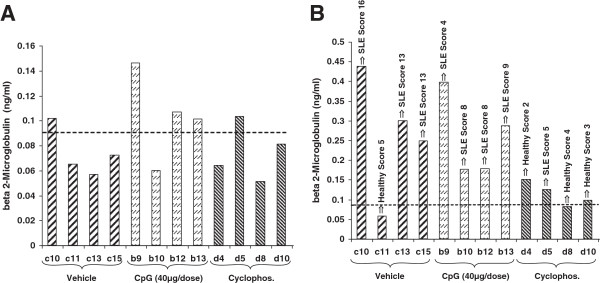
Beta2-microglobulin in urinary mouse samples at week 11 (A) and after the treatment (B).

Beta2-microglobulin in urinary mouse samples treated with vehicle, CpG and cyclophosphamide was determined at week 11 (Figure 7A) and at the end of the treatment (Figure 7B). A threshold of 0.1 ng/ml β2M in urine was selected for prediction of nephritis. For each group, urine samples were collected from mice with the highest renal activity at the end of the treatment.

Of the mice with upcoming nephritis, there were 7 of 10 subjects identified at week 11 with a significantly higher level of β2M in urine at the beginning of the treatment which develop nephritis at weeks 18–20. This is a probability of 70% compared to a prediction rate of only 10% by measurement of the proteinuria level alone (Table 3). The THG excretion in the urine of these mice was significantly lower than that of the other mice without nephritis. As shown in Figure 7B, the urinary β2M in mice at the end of the treatment was much higher than that in normal controls.

**Table 3:** Overview of ELISA results from the beta2-microglobulin determination

**Week**	**Disease state predicted on the urinary β2M level**^**a **^**(%)**	**Disease state predicted on the proteinuria level**^**a **^**(%)**
11	70	10
13	60	20
15	90	60
17	90	80
End of treatment	100	100

^a^Threshold of β2M in urine = 0.1 ng/ml and that of proteinuria level = 1 mg/ml.

### Conclusions

Since the 1970s, a variety of experimental techniques have been employed to identify urinary biomarkers of renal injury or kidney failure. While these approaches have met with some success, modern proteomic tools, such as MALDI, SELDI, LC-MS/MS, and MudPIT, now permit broad-based high-throughput analysis of the urinary proteome. Using the 2DE-PAGE approach in combination with ESI-MS, comparative urinary and kidney protein profiling was performed in a MRL-lpr/lpr mouse model of membranoproliferative glomerulonephritis. Paired kidney samples were analyzed at a late stage of LN disease with a high proteinuria level and healthy control mice. As a result, distinct urinary proteins were identified, of which several were differently expressed in the kidneys of the MRL-lpr/lpr mice. Most differently expressed proteins were identified and further confirmed by Western blot, enzyme-linked immunosorbent assay (ELISA) and RT-qPCR in kidney and urine samples. Two identified proteins were markedly altered in the kidney as well as in the urine compared to the healthy controls with a proteinuria level as low as 50 mg/dl: Tamm-Horsfall glycoprotein (THG) (4-fold lower in the urine) and β2M (3-fold higher in the urine). Western blots demonstrated a marked increase in the expression of β2M and a reduction of THG expression in the urine of the MRL-lpr/lpr mice, which correlated with the severity of disease. These findings may be related to early complement-dependent alterations in tubular protein expression which may play critical roles in the development of tubulointerstitial disease and provide experimental support for the use of urinary proteomic profiling of renal injury and/or kidney failure.

#### Beta2-microglobulin

Results from the 2DE-PAGE experiments on the kidney from MRL-lpr/lpr mice at the late stage of disease suggest that measurements of β2M in urine may represent a quantitative and predictive biomarker of *in vivo* polyclonal B cell activation. Our findings showed that an increase in urinary β2M occurred in MRL-lpr/lpr mice during intervals that in most cases preceded (70%–90%) 4–8 weeks, i.e. before the first symptomatic signs of nephritis (proteinuria) were visible. These results suggest that a sizeable lead time may exist before the occurrence of immunopathologic tissue damage in the kidney. In the course of the study, the measurement of urinary β2M was accomplished by a direct method using a solid-phase nanogram-sensitive ELISA assay that directly measures rat/mouse β2M in urine. As a control for the assessment of renal tubular function and the excretion of high molecular weight proteins in urine, long-term measurements of THG and proteinuria level were made by using WB and the Bradford technique, respectively, and compared with the β2M level. In addition to our findings, we assumed that longitudinal levels of urinary β2M may be used to track or monitor the *in vivo* immunopathologic B cell activity and may be helpful in predicting a disease relapse in human SLE patients.

#### THG

Our results suggest that the urinary excretion of THG, together with β2M, is a good indicator of the severity of renal inflammation and has therapeutic implications in monitoring the disease activity in mice with SLE. Because β2M is a potent amplifier of acute inflammation [19] and is rarely found in normal urine (as shown in Figure 7A), we measured this molecule in urine as an indicator of renal inflammation. Also, previous investigations [20] have demonstrated the usefulness of urinary THG and β2M measurement in lupus nephritis. We determined the urinary THG, proteinuria and β2M excretion in three groups of MRL-lpr/lpr mice before and after treatment with CpG, vehicle and cyclophosphamide. As shown in Figure 7A,B, the urinary β2M excretion increased only minimally after treatment in three of the four mice in each group. For each group of 10 animals, urine samples from the four mice with the highest renal activity at the end of the treatment were selected for further β2M determination. Three of the four mice in each group remained healthy and never reached a level significantly greater than 0.12 ng/ml, suggesting that the urinary excretion of β2M seems to be a good predictive indicator of the severity of renal inflammation and can have therapeutic implications in monitoring the disease activity in MRL-lpr/lpr mice. On the other hand, the urinary excretion of THG in mice with a higher proteinuria level was significantly lower than that in normal or in cyclophosphamide-treated subjects.

## Expert recommendations

In conclusion, the measurement of daily or weekly urinary THG and β2M excretion is a more practical method for monitoring the progression of lupus nephritis as compared with proteinuria or serial kidney biopsy alone as seen in Figures 6 and 7. β2M and THG can be used as sensitive indicators reflecting proximal and distal renal tubular damage in MRL-lpr/lpr mice with SLE or nephritis. The validation of our findings in corresponding human samples is ongoing and will be reported.

## Abbreviations

β2M: beta2-microglubulin; SLE: systemic lupus erythematosus; THG: Tamm-Horsfall glycoprotein.

## Competing interests

The authors declare that they have no competing interests.

## Authors' contributions

MB carried out the Western blot analysis. RH conceived of the study, performed the animal studies, participated in the design and coordination of the study and helped draft the manuscript. SH carried out the molecular genetic studies and the sequence alignment by RT-qPCR. JK participated in the design of the study and performed the statistical analysis. KK conceived the study, designed and coordinated the project and contributed to the writing of the manuscript. All authors read and approved the final manuscript.
